# Correlations between non-suicidal self-injury and problematic internet use among Chinese adolescents: a systematic review and meta-analysis

**DOI:** 10.3389/fpsyt.2024.1408508

**Published:** 2024-07-29

**Authors:** Xubin He, Qinyao Yu, Jing Peng, Jianghong Yu, Taiying Wu, Yuan Qin, Shengjun Wang, Tiaoxia Dong, Yulong Liao, Chunbi Hu, Ping Yang, Bo Yang

**Affiliations:** ^1^ Chongqing Mental Health Center, Chongqing, China; ^2^ Chongqing Medical School, Chongqing, China; ^3^ Chongqing jiangbei second hospital, Chongqing, China

**Keywords:** non-suicidal self-injury, problematic internet use, adolescents, China, meta-analysis

## Abstract

**Background:**

Non-Suicidal Self-Injury (NSSI) has continued to be a major issue for public health worldwide, especially among teenagers. Studies have found a certain correlation between NSSI and Problematic Internet Use (PIU). However, this relationship is still unclear among Chinese adolescents, a specific population. Hence, a meta-analysis was carried out on observational studies to explore the connection between NSSI and PIU in Chinese teenagers, aiming to provide more clarity on the correlation.

**Methods:**

To identify the link between NSSI and PIU, we scoured seven digital repositories until November 16, 2023. Employing a random-effects meta-analysis framework, we delved into the association between NSSI and PIU. Additionally, we carried out subgroup evaluations to scrutinize variables including geographical location, age demographics, research methodology, diagnostic instruments, gender, and variables controlled for confounding, like symptoms of depression. For amalgamating data, STATA software (version 16) was deployed.

**Results:**

In this analysis, we included 15 research papers encompassing a collective sample of 137,166 individuals. Our findings revealed a significant positive association between NSSI and PIU within the adolescent population in China, with an Odds Ratio (OR) of 2.02 and a 95% Confidence Interval (CI) ranging from 1.73 to 2.37. Notably, this correlation was markedly stronger in specific subgroups: adolescents from China’s Western regions exhibited an OR of 4.22 (95% CI: 3.44, 5.18); middle school attendees had an OR of 2.09 (95% CI: 1.92, 2.28); those diagnosed with concurrent depression disorders showed an OR of 2.32 (95% CI: 1.98, 2.73); and female adolescents demonstrated an OR of 2.49 (95% CI: 2.26, 2.75), highlighting the nuanced dynamics of this relationship.

**Conclusion:**

This meta-analysis indicates that PIU among adolescents is associated with an increased risk of NSSI. Our findings underscore the importance of targeting specific populations, including those in the western region of China, middle school students, adolescents with comorbid depression disorders, and female adolescents, who may be at higher risk of PIU and subsequently NSSI. These results emphasize the need for tailored interventions and preventive strategies to address these intertwined issues effectively.

**Systematic review registration:**

PROSPERO, identifier CRD42024496579.

## 1 Introduction

Non-Suicidal Self-Injury (NSSI) refers to deliberate and direct acts of harming oneself without the intent to die, such as cutting wrists, scratching arms, punching walls, taking drugs to experience pain, or pulling out hair (1). Previous studies often considered NSSI as a symptom of borderline personality disorder. In the most recent version of the Diagnostic and Statistical Manual of Mental Disorders (DSM-V), it is suggested that NSSI should be classified as a separate condition. Its purpose is to relieve interpersonal difficulties, unwanted thoughts, and emotions, as well as the negative impact on life (1). Research has shown that NSSI often begins in early adolescence, peaks in mid-adolescence, and declines in late adolescence (2). NSSI has become a major mental health issue among adolescents worldwide. In the past two decades, the number of individuals engaging in this behavior is rapidly increasing globally, particularly among adolescents (3). According to a study carried out by the Centers for Disease Control and Prevention in the US with 64,671 teenagers from the community, the occurrence of NSSI among adolescents varies from 6.4% to 30.8% (4). Similarly, a survey of 12,068 community adolescents across 11 European countries found a lifetime prevalence of NSSI ranging from 17.1% to 38.6% (5). Additionally, a meta-analysis found that 22.0% of adolescents worldwide have engaged in NSSI at some point in their lives, with an annual prevalence of 23.2% (6). In Asian countries, the overall lifetime prevalence of NSSI is higher at 32.6% compared to Western countries at 19.4% (7). Although NSSI does not involve suicidal intent, it frequently happens alongside suicidal behavior and can be a significant indicator of future suicide attempts and completed suicides (8). A meta-analysis of longitudinal studies estimated that individuals engaging in NSSI are 4.27 times more likely to attempt suicide and 1.51 times more likely to die by suicide subsequently (9). According to interpersonal psychological theory and the three-step theory of suicide behavior, NSSI is considered a “gateway” behavior to suicide (10). Repeated NSSI may desensitize individuals to pain, injury, fear, and death, thereby increasing the likelihood of suicide attempts. Therefore, it is essential to assess the factors influencing NSSI to prevent suicidal thoughts and behaviors.

In recent times, with the widespread adoption of the internet, it has evolved into an essential tool for adolescents across various domains including education, social interaction, and recreational activities (11). According to the 49th Statistical Report on Internet Development in China published by the China Internet Network Information Center (CINIC), the number of internet users in China has surged to 1.03 billion, with adolescents constituting 137 million of this demographic, accounting for 13.3% of the total internet user base in the country (12). Despite the manifold benefits that the internet offers to adolescents in terms of learning and daily life, excessive and uncontrolled usage of this technology may lead to what is commonly referred to as “Problematic Internet Use (PIU).” (13–15). PIU is defined as internet usage that results in psychological, social, educational, and/or occupational difficulties in an individual’s life, which encompasses terms such as “pathological internet use”, “excessive internet use”, “internet dependency”, “excessive smartphone usage”, and “compulsive internet use.” (16). Research suggests that PIU among adolescents can result in a myriad of severe issues, including attention deficits (17), academic underachievement (18), interpersonal conflicts (19, 20), family discord (21), anxiety (22), depression (23), and even instances of NSSI (24).

Adolescent NSSI arises from a complex interplay of social, psychological, and environmental factors (25). Notably, a substantial correlation exists between adolescent NSSI and PIU (26), with certain studies suggesting that PIU serves as a predictor for NSSI occurrences (24). However, a study by Liu et al. discovered that, upon controlling for depression variables, the linkage between adolescent NSSI and PIU dissipated (27). This finding underscores the intricate and contentious nature of the relationship between adolescent NSSI and PIU, with variations in outcomes across different research endeavors. To date, five systematic reviews have delved into this correlation, with two being narrative reviews (28, 29). While these reviews hint at a connection, methodological limitations obscure the true nature and strength of this association. Similarly, a meta-analysis spanning multiple European and Asian nations suggests that, even after adjusting for potential confounders like depression, PIU retains a link with heightened suicidal tendencies, albeit without a detailed exploration of the relationship between NSSI and PIU (30). Moreover, previous investigations have failed to specifically hone in on adolescents as a distinct cohort. In two systematic reviews conducted in China, the association between adolescent NSSI and PIU emerged as particularly robust. Nevertheless, these reviews encompassed a mere 3 and 4 articles, respectively, without delving deeper into potential influencing factors. Hence, further research is imperative to enhance the robustness of these findings (26, 31). Consequently, this study aims to amalgamate prior research on the NSSI-PIU relationship among Chinese adolescents, while considering factors such as measurement tool diversity, study type disparities, sample demographics (age, gender, region), and the control of potential confounders like depression, which could impact the outcomes of meta-analysis (30). Such an endeavor seeks to furnish a framework for the development of more targeted intervention initiatives in the future.

## 2 Methods

### 2.1 Materials and methods

The research relies on the PRISMA protocol for Systematic Reviews and Meta-Analyses (32). The protocol has been officially recorded in PROSPERO under registration number CRD42024496579.

### 2.2 Search strategy

In examining the correlation between NSSI and PIU, this investigation scoured through seven extensive databases, comprising the China National Knowledge Infrastructure, Wanfang Database, Chinese Biomedical Literature Service System, China Science and Technology Journal Database, PubMed, Embase, and Web of Science. The search spanned from the inception of these databases up to November 16, 2023. Employing a comprehensive approach, search terms were meticulously deployed across titles, abstracts, and subject terms, organized into three distinct keyword clusters (“non-suicidal self-injury,” “problematic internet use,” “adolescent”). Furthermore, efforts were made to contact authors to request missing data or information not explicitly mentioned in the articles. Additionally, manual perusal of article reference lists was undertaken to ensure the inclusivity of the literature. The encompassed publications spanned articles presented in both Chinese and English languages. For an in-depth understanding of the search strategy employed, please refer to (**Supplementary Material 1**).

### 2.3 Study selection criteria

The criteria for inclusion encompassed: (1) research of various types such as cross-sectional studies, case-control studies, and cohort studies; (2) populations comprising Chinese adolescents aged between 10 and 24 years (33); (3) literature elucidating the connection between NSSI and PIU while presenting pertinent Odds Ratio (OR) values; (4) publications available in either Chinese or English languages.

On the contrary, exclusion criteria encompassed: (1) study types involving reviews, conference abstracts, comments, and case reports; (2) literature lacking extractable data; (3) duplicate publications, with precedence given to those with the largest sample size.

### 2.4 Data extraction

To streamline and eliminate duplicate entries from the collected literature, NoteExpress served as the tool of choice. The screening process and quality evaluation were carried out independently by two researchers (XBH, YLL), adhering strictly to predefined inclusion and exclusion criteria. QYY spearheaded the data extraction process, with meticulous oversight from PY to guarantee precision. A thorough cross-verification of the literature was conducted, with allocation to a third investigator (BY). Extracted data encompassed details such as author(s), publication year, geographical region, study methodology, sample size, age demographics, effect size (OR) along with its 95% confidence interval, measurement instruments employed, and Adjusted for depression.

### 2.5 Quality assessment

XBH and YLL, two researchers, each performed a thorough assessment of cross-sectional study publications based on the standards established by the Agency for Healthcare Research and Quality (AHRQ). Discussion was used to resolve any inconsistencies. These evaluation criteria covered 11 key aspects, including the reliability of data sources, selection and definition of variables, determination of the time frame, representativeness of the sample, potential biases, statistical analysis methods employed, reliability of data collection procedures, and attention to subsequent quality assessments. Every element was classified as either ‘yes,’ ‘no,’ or ‘unclear.’ A value of 1 was given for ‘yes,’ while 0 was given for ‘no.’ The overall score varied from 0 to 11, with scores of 0 to 3 representing poor quality, 4 to 7 representing average quality, and 8 to 11 representing excellent quality (34). Only articles with a quality score greater than 4 were included in the meta-analysis. Furthermore, the quality of cohort studies was evaluated using the Newcastle-Ottawa Scale (NOS).The evaluation included eight components, focusing on three main aspects: subject selection, comparability among groups, and the precision of outcome or exposure measurement. Regarding subject selection, it included aspects such as the source of the sample, the recruitment process, and representativeness. We concentrated on the comparability between the study group and the control group in order to assess their similarity. The assessment of outcome measurement or exposure factor measurement examined the reliability and validity of data collection tools. Scores were assigned to each item on a scale of 0 to 9, with a cumulative score falling between 0 and 3 representing poor quality, 4 to 6 representing fair quality, and 7 to 9 representing good quality (35). Only articles with a quality score greater than 4 were included in the meta-analysis. The use of these assessment tools and criteria helped ensure a comprehensive evaluation and accurate judgment of the study quality.

### 2.6 Statistical analysis

Statistical analyses were carried out utilizing STATA software (version 16.0). The investigation into the relationship between adolescent NSSI and PIU involved the extraction of Odds Ratio (OR) values from each pertinent article. The culmination of this analysis resulted in the determination of the combined OR value along with its corresponding 95% confidence interval (95% CI). Heterogeneity was identified if the *P*-value was less than 0.05 or if the I-squared statistic (*I^2^*) exceeded 50% (36). Subgroup analyses were undertaken to probe into the potential sources of heterogeneity, categorized by region, age group, study design, measurement instruments, gender distribution, and control variables such as depressive factors. Intergroup disparities within these subgroups were evaluated utilizing a fixed-effects model. Moreover, sensitivity analyses were conducted to evaluate the influence of individual studies on the overall estimate. The significance level for the Egger test was established at *P* < 0.05 (37). To scrutinize for small-study effects, including those induced by publication bias, funnel plots (38) and Egger’s statistical test (39) were employed. Duval and Tweedie’s trim-and-fill method was implemented to estimate the overall effect size while addressing biases stemming from small-study effects (40). The threshold for statistical significance was set at *P* < 0.05.

## 3 Results

### 3.1 Characteristics of included studies and quality assessment

**Figure 1:**
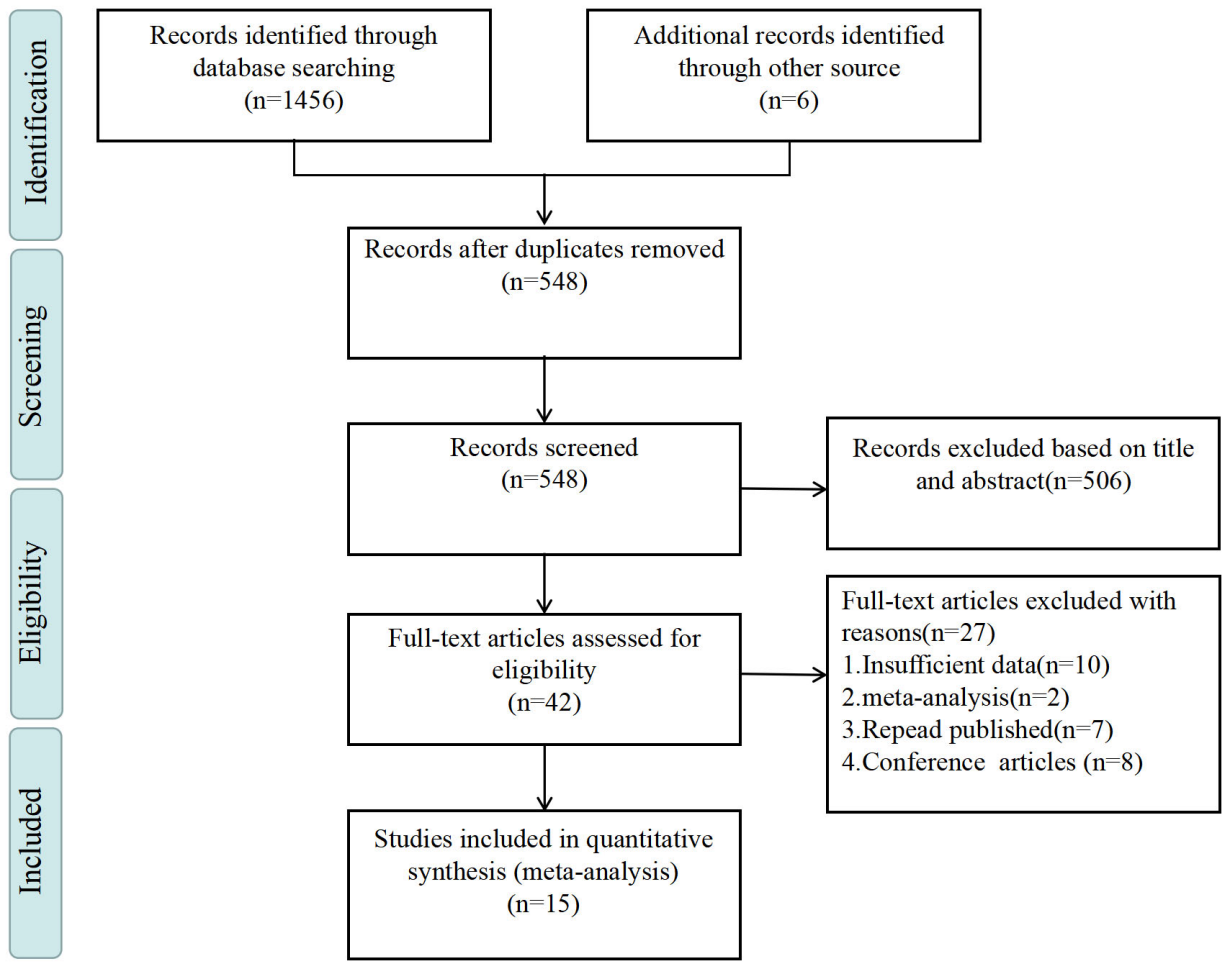
Literature selection process.

In accordance with the pre-established search strategy, a total of 1,462 articles were initially identified. Following the screening process facilitated by NoteExpress, 548 papers remained for further evaluation. Subsequently, through a meticulous examination of titles and abstracts, 42 articles were deemed pertinent and subjected to full-text scrutiny. Ultimately, 15 articles (24, 27, 41–53) met the inclusion criteria and were selected for comprehensive analysis. These studies collectively encompassed a participant pool of 137,166 individuals. Regarding the assessment tools employed for NSSI, 7 studies utilized validated instruments dedicated to self-harm measurement, while 6 studies utilized self-designed tools for the same purpose. Concerning PIU assessment, 7 studies utilized Young’s Internet Addiction Test developed by Young et al. (54), 3 studies employed the Chen Internet Addiction Scale by Chen et al. (55), and 3 studies utilized the Mobile Phone Addiction Index, initially translated into Chinese by Liu et al. (56). The remaining 2 studies utilized assessment tools developed by Tao et al. (57). The sample sizes across the included articles exhibited variance, ranging from 324 to 39,517 participants. Each included study underwent a comprehensive quality assessment, with five studies being categorized as high quality and the remaining ten as medium quality (**Supplementary Material 2**). **Table 1** provides an overview of the literature characteristics, while **Figure 1** delineates the progression of the inclusion process.

**Table 1:** Characteristics of the included article.

References	Region	Study types	Sample size (Male/Female)	Age range/Population	OR (*95%CI*)	Type of assessment (NSSI)	Type of assessment (PIU)	Adjusted for depression	Study quality scores
Lam et al., 2009 (41)	Eastern	Cross-sectional	1618(734/884)	M	1.68(1.15, 2.46)	SDSHQ	IAT	Yes	6
Duan et al., 2013 (42)	Eastern	Cross-sectional	39517(18991/20526)	M	1.31(1.18, 1.45)	CAHBSQ	MPAI	Yes	7
Tang et al., 2016 (43)	Eastern	Cross-sectional	5116(2777/2339)	16.23 ± 1.90/M	2.08(1.56, 2.59)	SDSHQ	SQAPMPU	No	6
Huang et al., 2016 (44)	Eastern	Cross-sectional	4822(2342/2480)	15.24 ± 2.36/M and C	1.53(1.14, 2.04)	CAHBSQ	IAT	Yes	6
Liu et al., 2017 (27)	Eastern	Cross-sectional	2479(1085/1494)	15.44 ± 0.61/C	1.38(0.97, 1.96)	QSSH	CIAS	Yes	6
Hsieh et al., 2018 (45)	Eastern	cohort	324(155/169)	22.1 ± 1.8/C	2.15(0.32, 14.56)	BSL-23	CIAS	Yes	6
Pan and Yeh 2018 (24)	Eastern	cohort	1507(1241/266)	15.93 ± 0.73/M	2.41(1.16, 4.99)	SDSHQ	CIAS	Yes	7
Cao et al., 2019 (46)	Eastern	Cross-sectional	2104(1075/1029)	M	1.65(1.21, 2.26)	SDSHQ	IAT	Yes	5
Li et al., 2019 (47)	multiple regions	Cross-sectional	22628(10990/11638)	15.36 ± 1.79/M	2.06(1.93, 2.20)	SDSHQ	SQAPMPU	No	5
Pang and Wang 2020 (48)	Eastern	Cross-sectional	14822(7648/7174)	15.27 ± 1.94/M	1.96(1.58, 2.41)	SDSHQ	IAT	No	6
Tang et al., 2020 (49)	Multiple regions	Cross-sectional	15623(8043/7580)	15.1 ± 1.8/M	1.95(1.64, 2.29) Male:1.47(1.22, 1.78) Female:1.86(0.95, 3.62)	CH-FASM	IAT	No	8
Wang et al., 2022 (50)	Western	Cross-sectional	2719(1246/1473)	13.42 ± 2.17/M and C	4.28(3.48, 5.27)	MASHS	MPAI	No	8
Qian et al., 2022 (51)	Western	Cross-sectional	1000(509/491)	M	3.87(2.24, 8.15)	OSI	IAT	Yes	6
Ma et al., 2023 (52)	multiple regions	cohort	1530(853/677)	12.9± 0.6/M	2.25(1.59, 3.19)	CH-FASM	IAT	Yes	8
Rong et al., 2023 (53)	Multiple regions	Cross-sectional	21357(10611/10746)	15.20 ± 1.79/M	2.37(2.19, 2.55) Male:2.23(2.07, 2.51) Female:2.51(2.30, 2.82)	CH-FASM	MPAI	No	8

NSSI, Non-suicidal self-injury; C, Cross-sectional studies; C, College; M, Middle school; CH-FASM, Chinese version of the Functional Assessment of Self-Mutilation; BSL-23, Borderline Symptom List; QSSH, Questionnaire for Suicidality and Self-Harm; CAHBSQ, Chinese Adolescent Health-related Behavior Survey Questionnaire; OSI, Ottawa Self-injury Inventory; SDSHQ, Self-Designed Self-Harm Questionnaire; MASHS, Modified adolescents self-harm survey; IAT, Internet Addiction Test designed by Young; CIAS, Chinese Internet Addiction Scale; SQAPMPU, Self-rating Questionnaire for Adolescent Problematic Mobile Phone Use; MPAI, Mobile Phone Addiction Index.

### 3.2 Homogeneity test and meta-analysis

**Figure 2:**
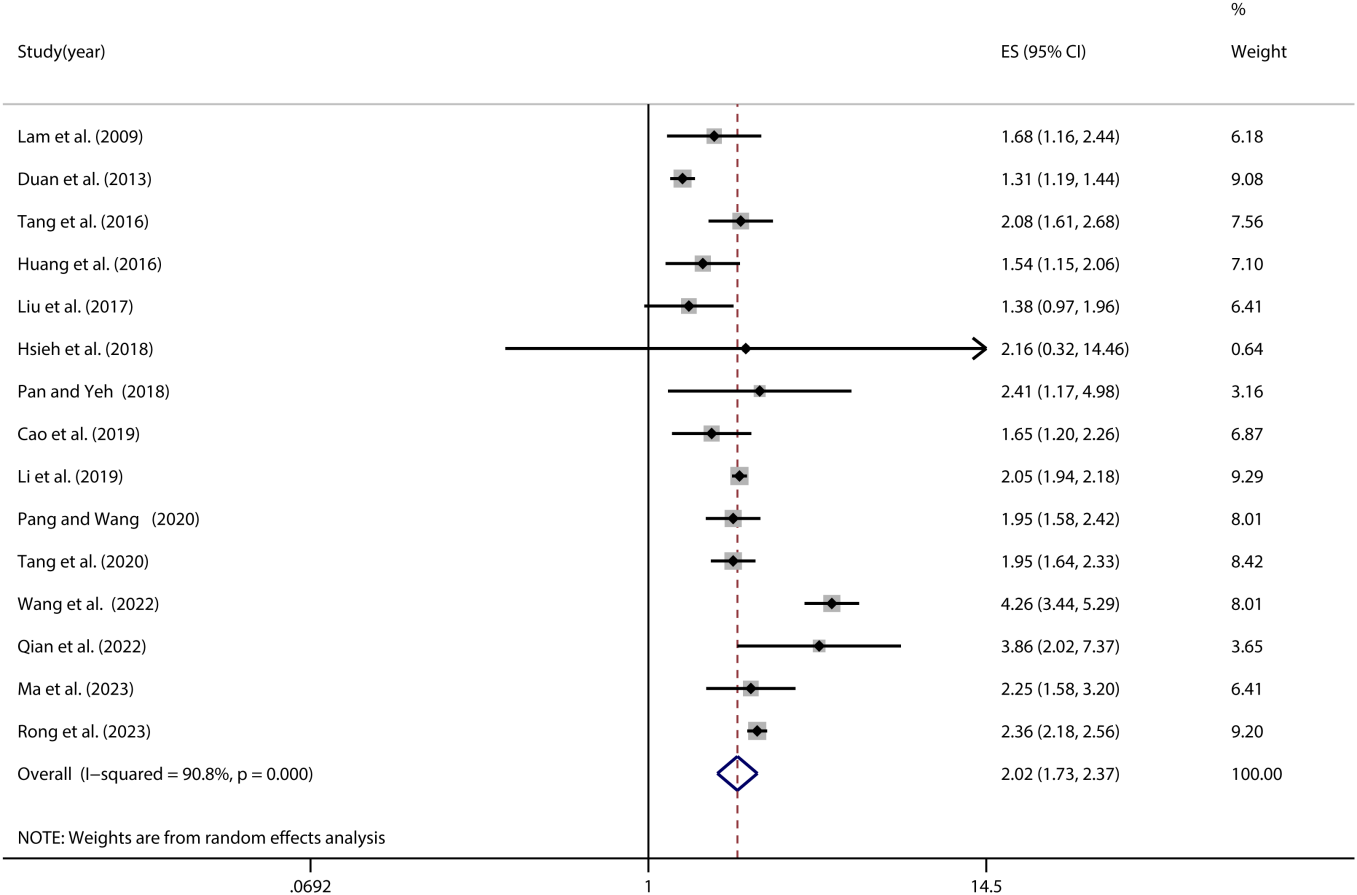
Forest plot of the relationship between NSSI and PIU.

Upon conducting homogeneity tests across the 15 publications encompassed in this study, notable heterogeneity was unveiled (*I^2 =^* 90.8%, *P* < 0.001). Utilizing the random-effects model, a pooled effect size concerning the association between NSSI and PIU was determined to be (OR=2.02, 95% CI: 1.73, 2.37). Moreover, the statistical analysis underscored a significant relationship between NSSI and PIU (*Z* = 8.72; *P* < 0.001) (refer to **Figure 2**).

### 3.3 Processing of the heterogeneity between NSSI and PIU

Significant heterogeneity was detected through the implementation of the random-effects model. Consequently, a subgroup analysis was undertaken to mitigate this observed heterogeneity.

### 3.4 Subgroup analysis

Subgroup analysis was conducted, exploring various factors associated with both NSSI and PIU, including geographical region, age, gender, study design, measures used for PIU and NSSI, and adjustment for depression (as outlined in **Table 2**).

**Table 2:** Subgroup analysis showing OR of NSSI for PIU.

Variables	95% Cl for OR		Heterogeneity test		Heterogeneity between the groups (*P*-value)
	*OR*	*95% Cl*	*I^2^* (%)	*P*-value	
**Region**					< 0.001
Eastern(n=9)	1.65	1.41, 1.95	63.80	0.005	
multiple regions(n=4)	2.15	1.95, 2.40	66.90	0.028	
Western(n=2)	4.22	3.44, 5.18	0.00	0.774	
**Age**					< 0.001
Middle school student(n=10)	2.09	1.92, 2.28	48.00	0.044	
College student(n=2)	1.40	0.99, 1.98	97.70	< 0.001	
Middle school and College students(n=3)	2.05	0.95, 4.42	0.00	0.648	
**Study design**					0.417
Cross-sectional (n =12)	1.99	1.68, 2.36	92.70	< 0.001	
Cohort (n=3)	2.27	1.66, 3.11	0.00	0.984	
**PIU measures**					0.213
IAT(n=7)	1.90	1.66, 2.17	32.10	0.183	
CIAS(n=3)	1.55	1.13, 2.12	0.00	0.373	
MPAI(n=3)	2.34	1.37, 4.02	98.50	< 0.001	
SQAPMPU(n=2)	2.06	1.94, 2.18	0.00	0.94	
**NSSI measures**					0.464
Validated measures tool(n=9)	2.11	1.58, 2.81	94.60	< 0.001	
SDSHQ(n=6)	2.03	1.92, 2.14	0.00	0.67	
**Adjusted for depression**					< 0.001
Yes(n=9)	1.72	1.41, 2.09	64.20	0.004	
No(n=6)	2.32	1.98, 2.73	89.50	< 0.001	
**Gender**					0.003
Male(n=2)	1.83	1.22, 2.73	92.60	< 0.001	
Female(n=2)	2.49	2.26, 2.75	0.00	0.383	

SDSHQ,Self-Designed Self-Harm Questionnaire; IAT, Internet Addiction Test designed by Young; CIAS, Chinese Internet Addiction Scale; SQAPMPU, Self-rating Questionnaire for Adolescent Problematic Mobile Phone Use; MPAI, Mobile Phone Addiction Index.

**Figure 3:**
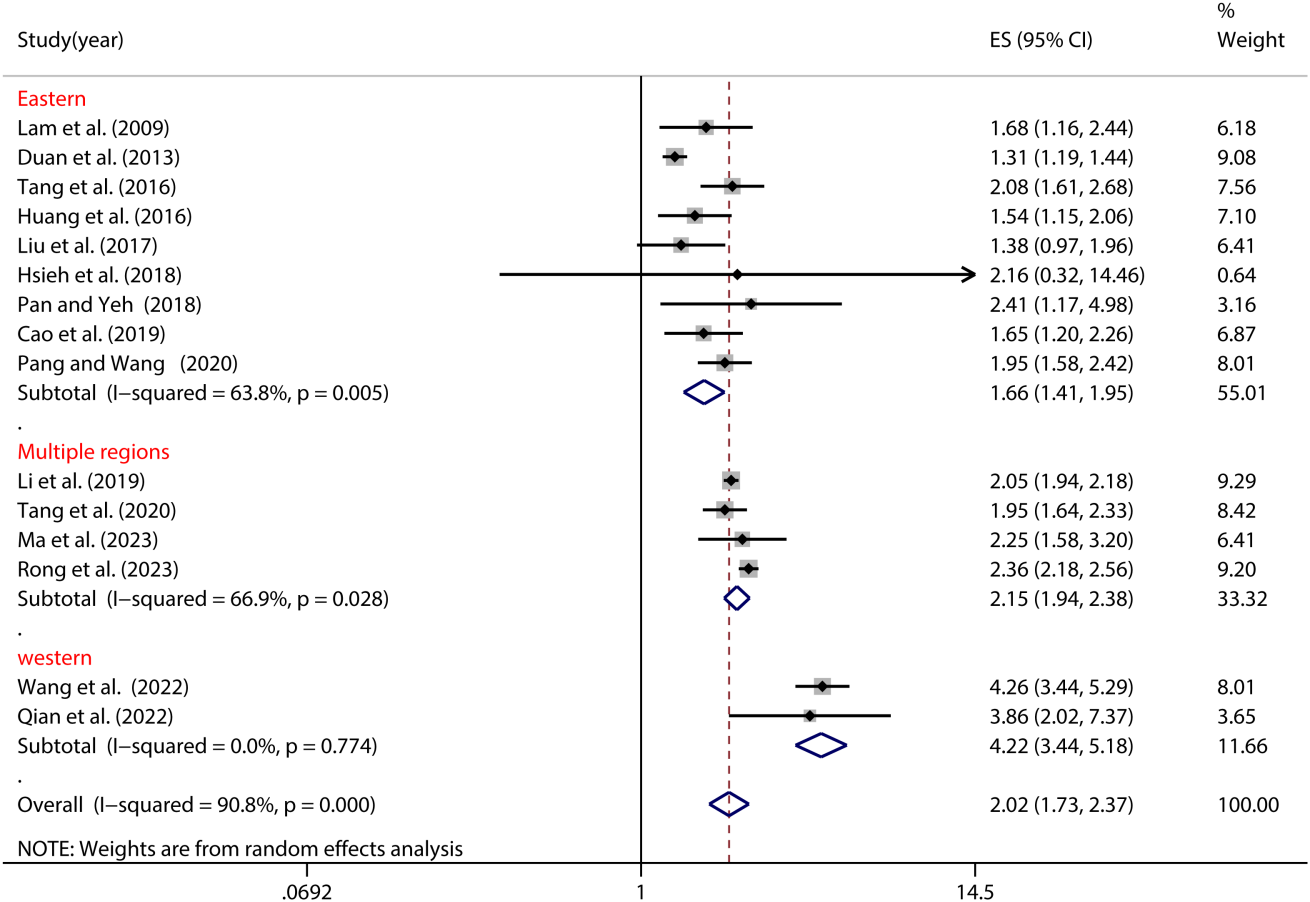
Moderation effect of regional differences on NSSI and PIU.

As depicted in **Figure 3**, significant regional disparities emerged as moderators of the NSSI-PIU relationship (see **Table 2**). Notably, adolescents hailing from the western region exhibited the highest vulnerability to NSSI among those grappling with PIU (OR=4.22, 95% CI: 3.44, 5.18). Conversely, the risk of NSSI appeared markedly lower among counterparts from the eastern region (OR=1.65, 95% CI: 1.41, 1.95) (refer to **Figure 3**).

**Figure 4:**
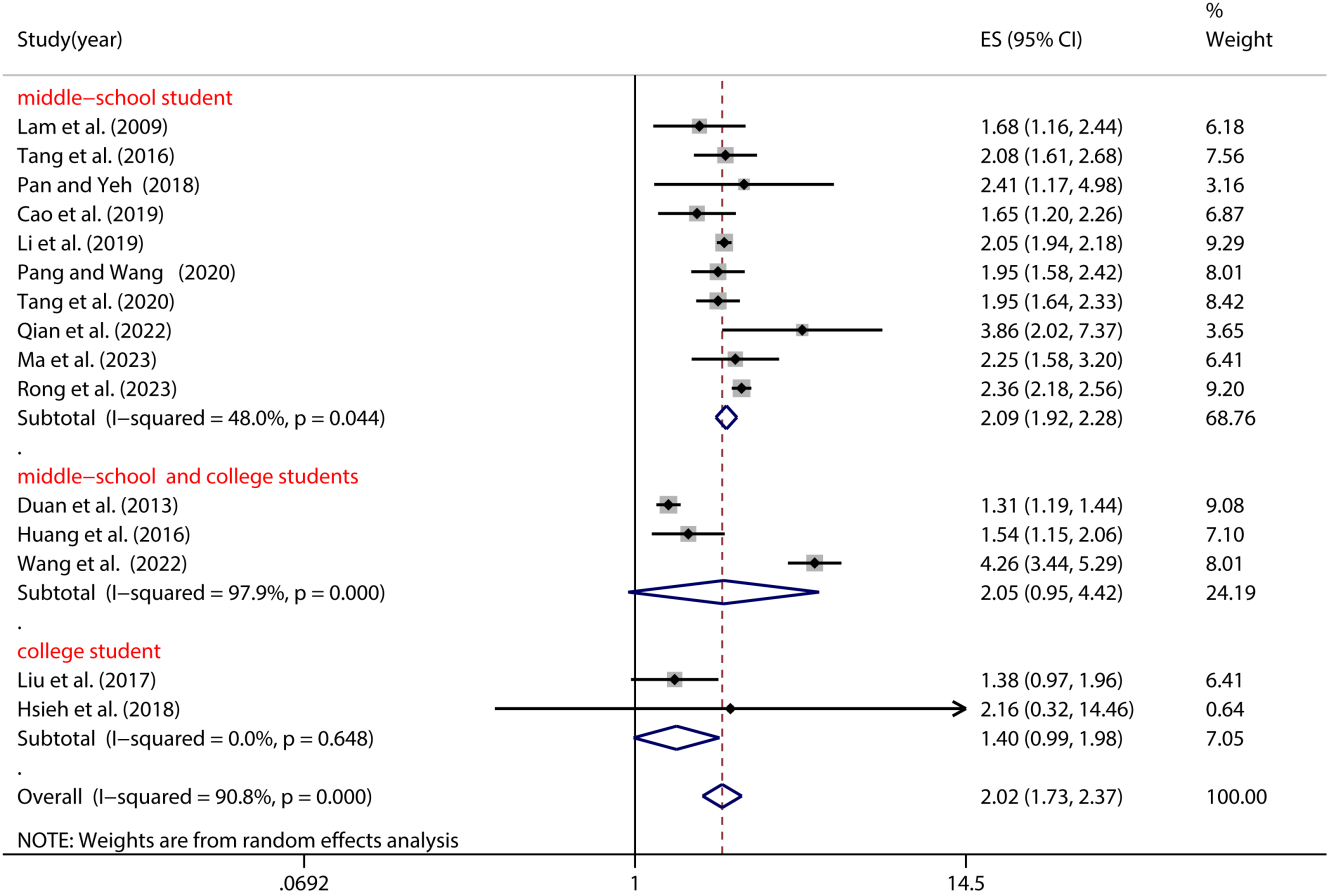
Moderation effect of age on NSSI and PIU.

Age, as showcased in **Figure 4**, also exerted a notable moderation effect on the NSSI-PIU association (as detailed in **Table 2**). Specifically, Chinese middle school students with PIU demonstrated a significantly heightened risk of NSSI (OR=2.09, 95% CI: 1.92, 2.28) compared to their college counterparts (OR=1.40, 95% CI: 0.99, 1.98) (see **Figure 4**).

**Figure 5:**
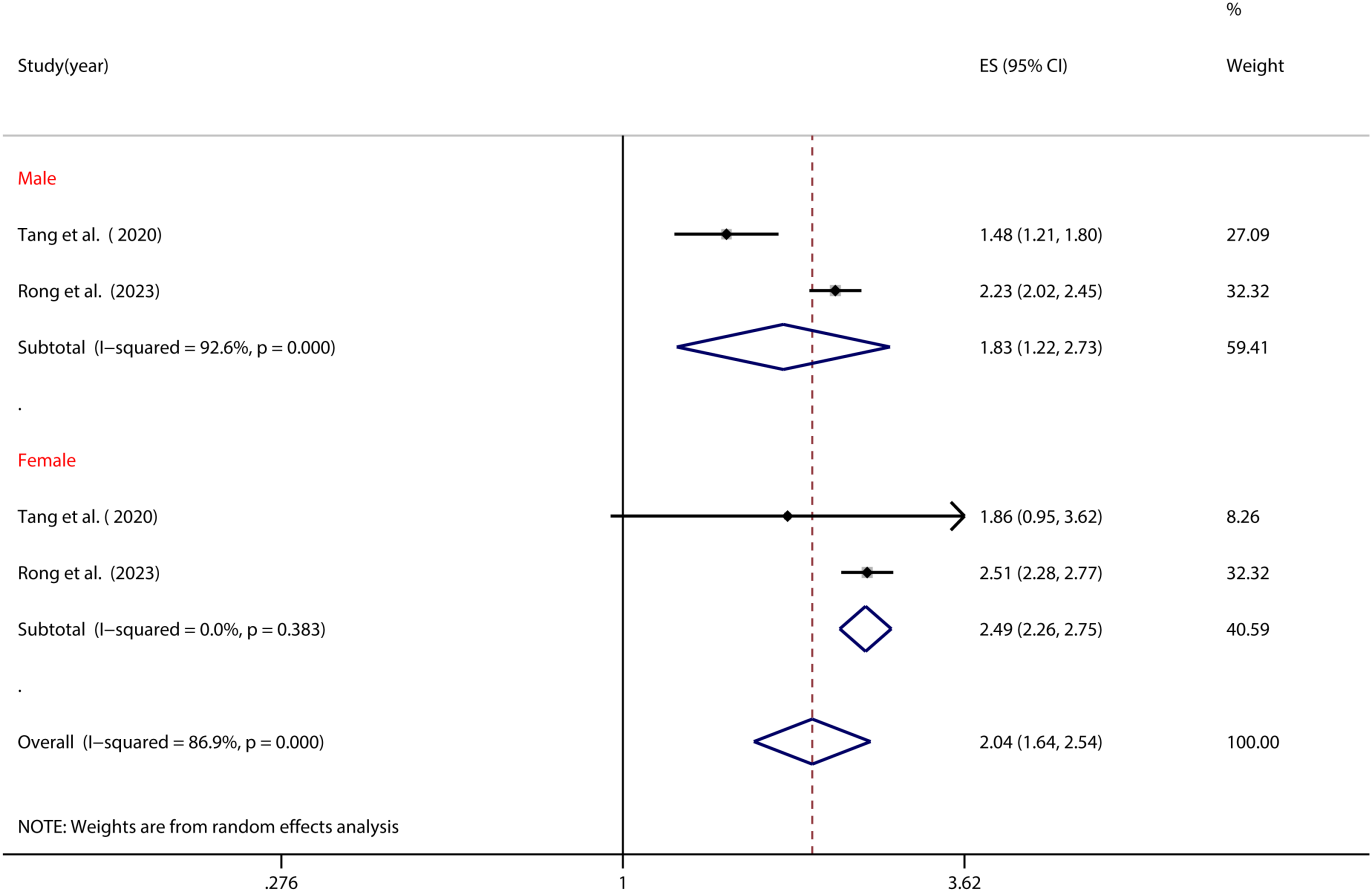
Moderation effect of gender on NSSI and PIU.

**Figure 5** underscores the profound gender-based modulation of the NSSI-PIU relationship (as elaborated in **Table 2**). Among Chinese adolescents, females grappling with PIU exhibited a markedly elevated NSSI risk (OR=2.49, 95% CI: 2.26, 2.75) compared to their male counterparts (OR=1.83, 95% CI: 1.22, 2.73) (refer to **Figure 5**).

**Figure 6:**
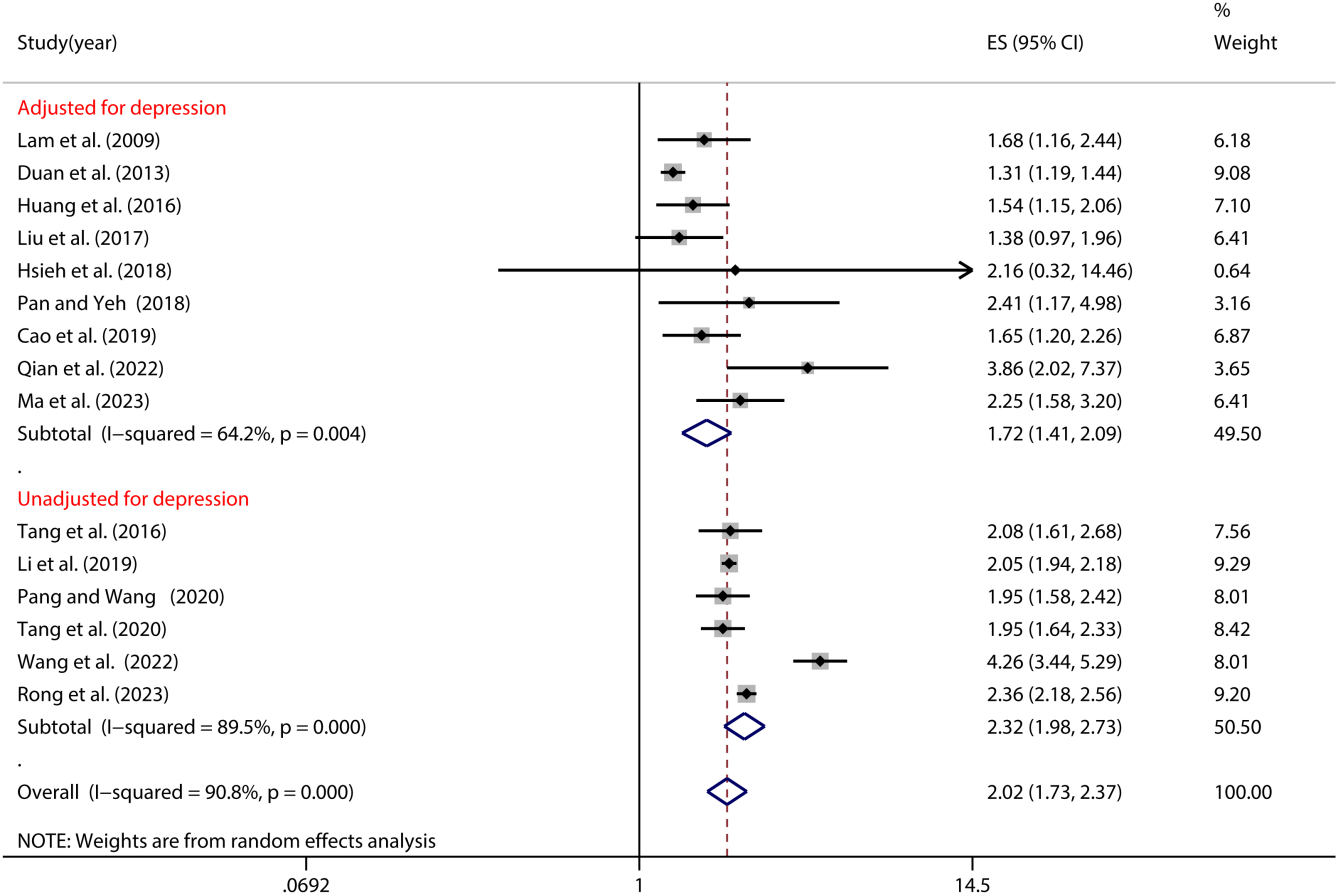
Moderation effect of depression control on NSSI and PIU.

Furthermore, as illustrated in **Figure 6**, the control for depression exerted a significant moderating influence on the NSSI-PIU correlation (as indicated in **Table 2**). Particularly noteworthy is the observation among Chinese adolescents, where the NSSI risk peaked when depression remained uncontrolled (OR=2.32, 95% CI: 1.98, 2.73). Conversely, the risk attenuated substantially when depression was effectively managed (OR=1.72, 95% CI: 1.41, 2.09) (see **Figure 6**).

### 3.5 Sensitivity analysis

**Figure 7:**
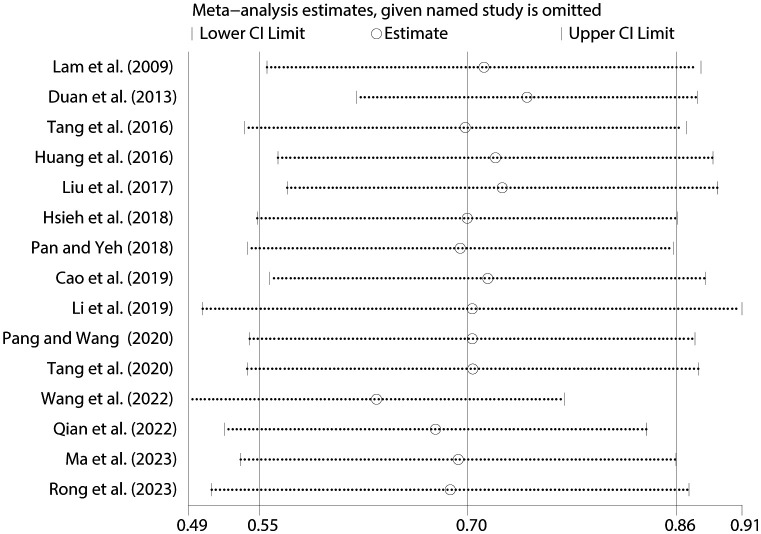
Sensitivity analysis between NSSI and PIU.

To gauge the reliability of our findings, a sensitivity analysis was meticulously performed, systematically eliminating individual studies and recalibrating the overall correlation coefficient between NSSI and PIU. Encouragingly, the sensitivity analysis revealed minimal fluctuations in the overall correlation coefficient, underscoring the stability of our results (refer to **Figure 7**).

### 3.6 Publication bias

**Figure 8:**
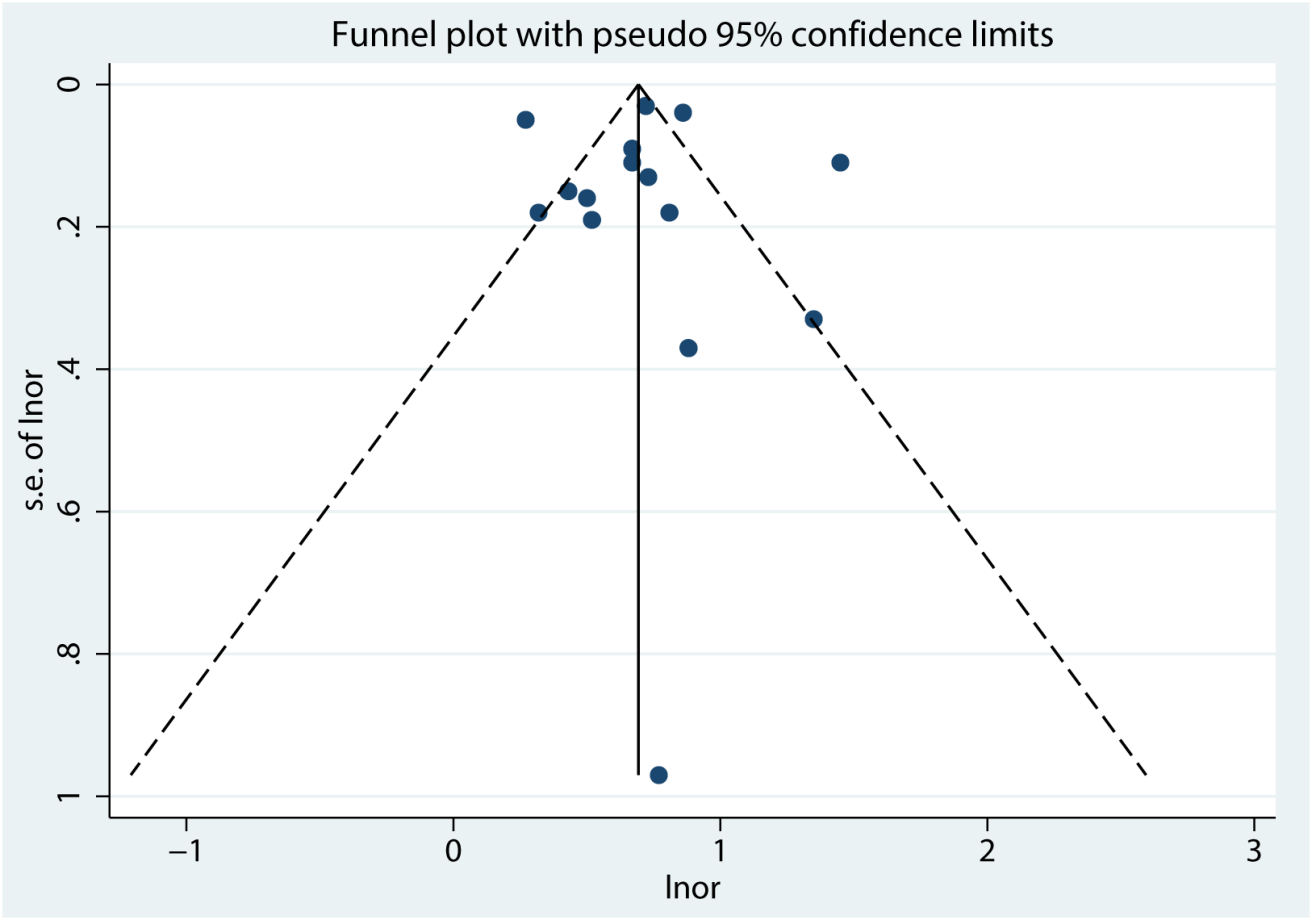
Funnel plot between NSSI and PIU.

**Figure 9:**
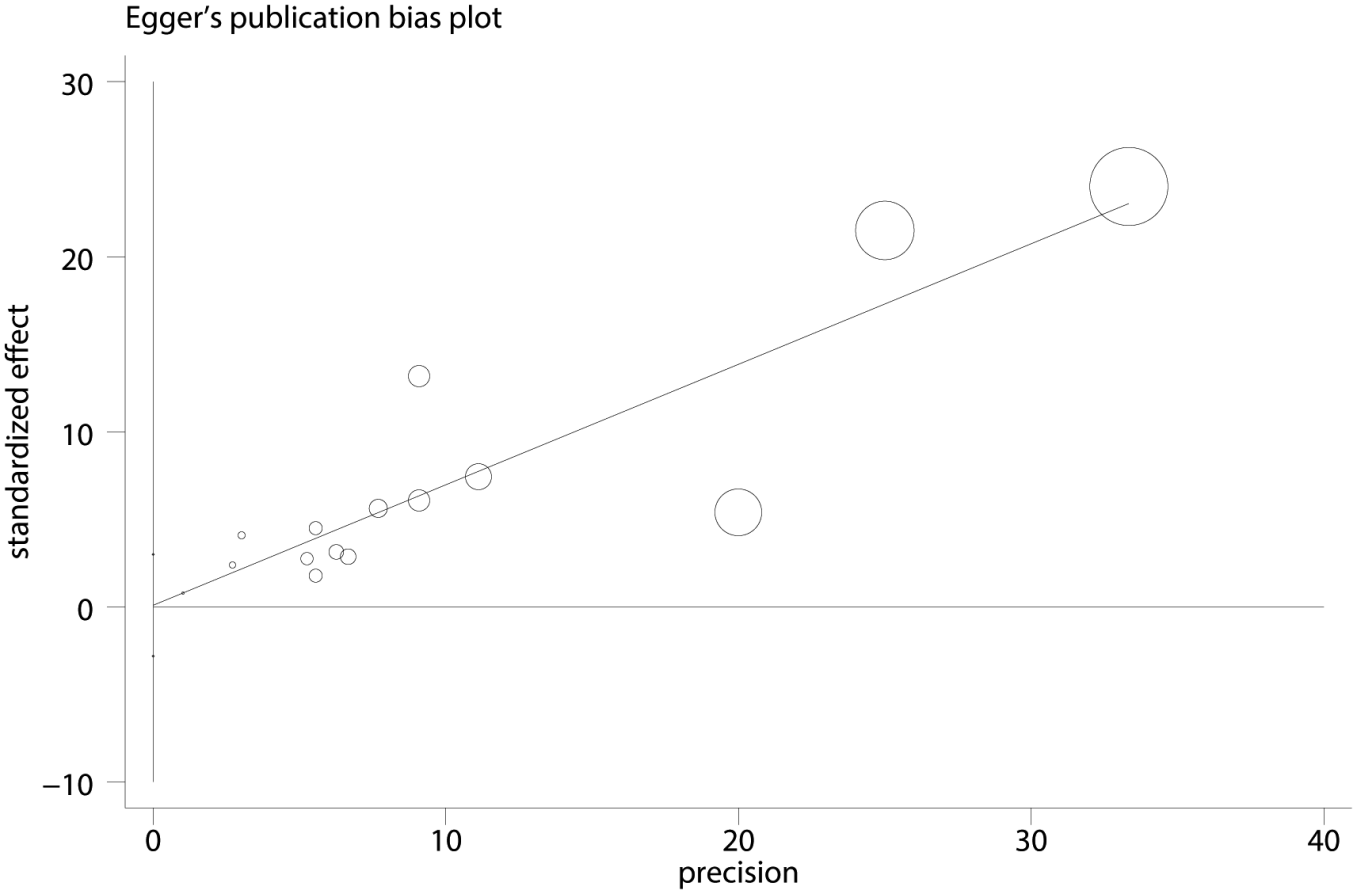
Egger plot between NSSI and PIU.

In evaluating the symmetry of the funnel plot, which assessed the overall correlation coefficient between NSSI and PIU (**Figure 8**), a subjective analysis revealed a balanced distribution on both sides. Moreover, the Egger regression test yielded a non-significant result (*t* = 0.06, *p* = 0.95) (**Figure 9**), providing further evidence of the absence of significant publication bias.

## 4 Discussion

In recent years, adolescent NSSI has emerged as a pressing global public health concern (58). Concurrently, PIU has garnered increasing attention as a significant contributing factor to problematic behaviors among youth (26). Despite extensive research efforts, a definitive consensus regarding the relationship between NSSI and PIU remains elusive (24, 27). The outcomes of this meta-analysis reveal a notable correlation between NSSI and PIU among Chinese adolescents, with those afflicted by PIU being over twice as likely to engage in NSSI compared to their non-PIU counterparts. This finding resonates with earlier research, including the study conducted by Fan et al. (26). The rationale behind this phenomenon can be dissected as follows: Firstly, a reciprocal interaction between NSSI and PIU may exist, wherein the internet serves as a platform for NSSI exposure. Adolescents immersed in PIU are more predisposed to encountering peers and information related to NSSI, thereby amplifying the likelihood of imitation and engagement in NSSI behaviors. Social reinforcement further accentuates the risk of NSSI occurrences, ultimately culminating in the manifestation of such behaviors (59). Secondly, both NSSI and PIU share addictive propensities (60, 61). The Interaction of Person-Affect-Cognition-Execution (I-PACE) model posits that addictive behaviors ensue from intricate interactions between susceptibility factors, emotional and cognitive responses to specific stimuli, and executive functions like inhibition control and decision-making. Throughout the addictive trajectory, the correlation between cue-reactivity/craving and diminished inhibition control fosters habitual behaviors (62). Research suggests that prolonged PIU may induce structural and functional alterations in the adolescent brain, particularly impacting areas pertinent to executive control and decision-making, thereby elevating the risk of NSSI (63–66). Additionally, sustained PIU could precipitate neuroadaptation alterations within the brain’s reward system. Moreover, addictive behaviors are associated with the release of hormones and neurotransmitters (e.g., endogenous opioids), potentially distorting individuals’ reward and punishment perceptions, consequently heightening their inclination towards NSSI (67, 68). Thirdly, PIU may engender social detachment from real-life interactions and undermine adolescents’ coping mechanisms for managing challenging emotions and sentiments. Adolescents engrossed in PIU may necessitate additional support and mechanisms to alleviate their psychological and emotional distress. The intense physiological sensations accompanying NSSI can serve as a diversion from internal psychological distress towards external physiological experiences, thereby instigating NSSI incidents (61, 69). Fourthly, PIU could precipitate personality disintegration, impairing adolescents’ typical social functioning and diminishing their vigilance and fear-related emotions. This often culminates in impulse inhibition disorders, thereby heightening the likelihood of NSSI occurrences (70). Lastly, adolescent PIU closely intertwines with mental health concerns such as anxiety and depression, both significant predictors of NSSI. PIU might initially function as a coping mechanism to alleviate mental health challenges; however, over time, it exacerbates these issues, thereby escalating the risk of NSSI (71, 72).

The study’s findings unveil a significant moderating impact of regional disparities on the relationship between NSSI and PIU. Notably, in China’s western region, adolescents grappling with PIU face a substantially heightened risk of NSSI, up to four times greater compared to several other regions in the country, with the lowest risk observed in the eastern region. This discrepancy may stem from the relatively underdeveloped economic status and prolonged parental absences due to occupational commitments prevalent in the western region. Such circumstances may foster an environment lacking in emotional communication within adolescents’ families, consequently elevating the risk of PIU and subsequent NSSI (73). Dysfunctional family dynamics, in turn, exacerbate the vulnerability to NSSI among adolescents (74). Thus, the association between NSSI and PIU appears more pronounced among adolescents in China’s western region, accentuating the imperative to fortify mental health intervention efforts targeted at this demographic.

Furthermore, the study reveals a significant moderation effect of age on the relationship between NSSI and PIU. Specifically, adolescents afflicted with PIU exhibit a heightened risk of NSSI compared to university students. This observation can be attributed to the pivotal stage of physiological and psychological development experienced by adolescents, characterized by nascent self-awareness, coping mechanisms, and social skills (75). In contrast, university students have transitioned into a more autonomous and mature phase of life. Owing to adolescents’ relatively weaker self-regulation and emotional control capabilities, they are more susceptible to the adverse effects of PIU, consequently escalating the risk of NSSI (76, 77). Additionally, adolescents are particularly influenced by peer dynamics and social networks, rendering them more susceptible to PIU. Conversely, university students may possess superior critical thinking and self-regulation abilities, which could mitigate PIU’s detrimental impact on their mental well-being, thus lowering the risk of NSSI (78, 79). Furthermore, adolescents often encounter barriers in accessing mental health resources and may feel disinclined to seek assistance due to familial, academic, and societal pressures (80). In contrast, university students typically have greater access to diverse mental health support systems and exhibit a greater willingness to seek aid, thereby reducing the likelihood of NSSI. This underscores the significance of addressing PIU concerns among adolescents and furnishing them with tailored support and intervention measures to mitigate NSSI occurrences.

Additionally, the study identifies a significant moderating influence of controlling for depression variables on the NSSI-PIU relationship. This implies that the heightened risk of NSSI among adolescents with PIU may be partially mediated by depression, although other contributing factors are also at play. This finding aligns with the conclusions drawn by Cheng et al. (30). One plausible explanation is that depression disorders are inherently intertwined with PIU; adolescents grappling with depression are more susceptible to PIU as a coping mechanism to alleviate psychological distress, with PIU potentially exacerbating depressive symptoms, thus perpetuating a detrimental cycle that heightens the risk of NSSI (81, 82). Moreover, adolescents contending with depression often grapple with heightened social isolation and a dearth of effective coping mechanisms, rendering them more susceptible to immersing themselves in the online realm, consequently elevating the risk of PIU and, by extension, the incidence of NSSI (83). Henceforth, it is advisable to screen adolescents with problematic Internet use for NSSI risk, even in the absence of depression, as additional factors may heighten NSSI susceptibility among individuals grappling with PIU.

Similarly, gender was identified as a significant moderator in the relationship between NSSI and PIU in this study. Findings indicate that among those afflicted with internet addiction, female adolescents are more predisposed to NSSI occurrences. This observation could be elucidated by several factors. Firstly, female adolescents tend to share personal experiences and emotions more openly on social media platforms, rendering them more susceptible to external judgments regarding their appearance, body image, and lifestyle choices. Such online interactions may exacerbate issues related to self-identity and self-doubt, thereby heightening the risk of NSSI subsequent to PIU engagement (84, 85). Secondly, female adolescents are more prone to internalizing problems such as depression and anxiety, while male adolescents are inclined towards externalizing problems such as aggression and behavioral issues. Among adolescents grappling with PIU, internalizing problems are more likely to manifest in females, consequently augmenting their NSSI vulnerability (86, 87). Thirdly, female adolescents typically rely more heavily on social support networks than males, and may lack effective self-regulation skills when confronted with stressors. This renders them more susceptible to immersing themselves in the online realm as a means of escaping real-life challenges, thereby escalating the risk of NSSI (88, 89). It’s important to note, however, that the subgroup analysis in this study comprised only two articles, potentially limiting the generalizability of the findings.

However, neither the assessment tools for PIU nor those for NSSI exhibited a moderating effect on the relationship between NSSI and PIU. This suggests a degree of stability and consistency in the outcomes derived from various assessment instruments. It should be noted that the inclusivity of PIU encompasses various terms and employs different measurement tools, which may limit the generalizability of research findings. Therefore, further exploration is needed to determine whether the relationship between PIU and NSSI is moderated by other measurement instruments. Lastly, different study methodologies also failed to moderate the relationship between NSSI and PIU, indicating that disparate study designs do not significantly influence the study outcomes.

### 4.1 Advantages and limitations

This review meticulously gathered and analyzed published data to uncover the connection between NSSI and PIU among Chinese adolescents. The inclusion of studies with sizable sample sizes and diverse participant demographics greatly strengthened the credibility of our research findings. Insights gleaned from this investigation have the potential to provide new perspectives for the prevention and clinical management of NSSI among Chinese adolescents struggling with PIU. Thus, they furnish a solid evidential foundation for practice and intervention in this domain.

However, several limitations warrant acknowledgment. Primarily, the predominant inclusion of cross-sectional observational studies in our analysis, with only a sparse inclusion of longitudinal studies, signifies that our findings denote a correlation between NSSI and PIU rather than establishing a causal relationship. Secondly, our study exclusively focused on Chinese adolescents, which limited the generalizability of our findings by excluding research involving other age groups. Thirdly, only a few potential moderating variables were found to have significant effects, and a substantial portion of the observed heterogeneity remains unexplained. Therefore, future meta-analyses need to examine the role of potential new moderating factors in the correlation between PIU and NSSI. Additionally, we only included literature in Chinese and English and did not include unpublished literature, which may lead to publication bias in the results. Lastly, the use of self-report questionnaires in all studies does not equate to clinical assessment, and this study integrates different terms regarding internet use, which may affect the robustness of the results. Therefore, future research could further clarify the concepts related to internet use to establish more reliable conclusions from studies that relate these different uses with other variables of interest.

## 5 Conclusion

The outcomes derived from this systematic review and meta-analysis undeniably underscore a robust and statistically significant positive correlation between NSSI and PIU among Chinese adolescents, affirming that adolescent PIU heightens the propensity for NSSI occurrence. Particular emphasis should be placed on specific demographic segments, including those situated in the western regions of China, middle school attendees, adolescents grappling with concurrent depressive disorders, and female adolescents, as they may confront elevated rates of PIU, consequently amplifying their vulnerability to NSSI risk. Thus, the imperative for early detection and targeted interventions for PIU adolescents cannot be overstated, directly mitigating the frequency of NSSI incidents. Adolescence represents a pivotal developmental phase marked by distinct psychological and physiological attributes, during which educational institutions and familial environments play pivotal roles in reinforcing adolescent mental health education. This entails heightening awareness surrounding PIU and NSSI while bolstering adolescents’ mental health literacy and resilience. Concurrently, addressing PIU concerns among adolescents necessitates the establishment of tailored intervention frameworks. Educational institutions can institute dedicated platforms or curricula geared towards the prevention and management of PIU, offering psychological counseling and support services. Psychologists and clinical practitioners can devise individualized treatment regimens tailored to specific circumstances, aiding adolescents in cultivating constructive coping mechanisms, alleviating psychological distress, and mitigating NSSI occurrences. Moreover, encouraging adolescents to actively engage in extracurricular pursuits such as sports, arts, and cultural activities can foster diverse interests and hobbies, thereby curtailing excessive internet utilization. Promoting a wholesome lifestyle encompassing sound sleep patterns, balanced nutrition, and moderate physical activity is also advocated to alleviate PIU and NSSI tendencies. By adopting a multifaceted approach encompassing early intervention, targeted support services, and lifestyle modifications, stakeholders can effectively address the intertwined challenges posed by PIU and NSSI among adolescents, safeguarding their overall well-being.

## Data availability statement

The original contributions presented in the study are included in the article/**Supplementary Material**. Further inquiries can be directed to the corresponding authors.

## Author contributions

XH: Validation, Software, Project administration, Methodology, Investigation, Formal analysis, Data curation, Conceptualization, Writing – review & editing, Writing – original draft. QY: Writing – original draft, Data curation. JP: Writing – review & editing, Supervision, Methodology. JY: Writing – review & editing, Software. TW: Writing – review & editing, Investigation. YQ: Writing – review & editing, Software. SW: Writing – review & editing, Methodology. TD: Writing – review & editing, Methodology. YL: Writing – original draft, Data curation. CH: Writing – review & editing, Investigation. PY: Writing – review & editing, Validation. BY: Writing – review & editing, Validation, Supervision.
