# Supplementary material for: Correlations between non-suicidal self-injury and problematic internet use among Chinese adolescents: a systematic review and meta-analysis
Source: Front Psychiatry. 2024 Jul 29;15:1408508. doi: 10.3389/fpsyt.2024.1408508 (PMC11317390; doi:10.3389/fpsyt.2024.1408508)
Supplement: Supplementary file 1 [file Table_1.docx]

**Additional file**

**Supplementary material 1:** Search strategy used in the current systematic review and meta-analysis

**Supplementary material 2:** Methodological quality assessment of the literature

***PUBMED***

#1

(deliberate self harm[MeSH Terms]) OR (self injurious behavior[MeSH Terms]) OR (self-injury[MeSH Terms])

#2

(self-harm[Title/Abstract]) OR (self harm*[Title/Abstract]) OR (self-injur*[Title/Abstract]) OR (self-cut*[Title/Abstract]) OR (self-destruct[Title/Abstract]) OR (nonsuicidal self-injur*[Title/Abstract]) OR (non-suicidal self injur*[Title/Abstract]) OR (deliberate self harm [Title/Abstract]) OR (self-mutil*[Title/Abstract]) OR (overdos*[Title/Abstract]) OR (self-inflicted injur*[Title/Abstract]) OR (self inflicted injur*[Title/Abstract]) OR (para-suicid*[Title/Abstract]) OR parasuicid*[Title/Abstract] OR (suicidal behav*[Title/Abstract]) OR (self?harm*[Title/Abstract]) OR (self?injur*[Title/Abstract]) OR (self?poison*[Title/Abstract]) OR (self?inflict*[Title/Abstract])

#3

(Internet Addiction Disorder [Mesh])

#4

(cell phone[Title/Abstract]) OR (cell phones[Title/Abstract]) OR (cellular phone[Title/Abstract]) OR (cellular phones[Title/Abstract]) OR (cellular telephone[Title/Abstract]) OR (cellular telephones[Title/Abstract]) OR (mobile devices[Title/Abstract]) OR (mobile phone[Title/Abstract]) OR (smart phone[Title/Abstract]) OR (smartphone[Title/Abstract])

#5

(addiction[Title/Abstract]) OR (dependence[Title/Abstract]) OR (dependency[Title/Abstract]) OR (abuse[Title/Abstract]) OR (addicted to[Title/Abstract]) OR (overuse[Title/Abstract]) OR (problem use[Title/Abstract]) OR (compensatory use[Title/Abstract])

#6

(internet addiction [Title/Abstract]) OR (problematic internet use [Title/Abstract]) OR (internet addiction disorder [Title/Abstract]) OR (pathological internet use [Title/Abstract]) OR (excessive internet use [Title/Abstract]) OR (compulsive internet use [Title/Abstract]) OR (internet dependency [Title/Abstract]) OR (computer addiction [Title/Abstract]) OR (internet use disorder [Title/Abstract])

#7

(“journal article” [Publication Type])

#8

(RCT or “random controlled trial” [Title/Abstract])

(#1 OR #2) AND (#3 OR (#4 AND #5) OR #6) AND #7 NOT #8

N=139

***Web of Sci***

#1

TS=(“deliberate self harm” OR “self injurious behavior” OR “self-injury” OR “self-harm” OR “self harm*” OR “self-injur*” OR “self injur*” OR “self-cut*” OR “self-destruct” OR “nonsuicidal self-injur*” OR “non-suicidal self injur*” OR “deliberate self harm” OR “self-mutil*” OR overdos* OR “self-inflicted injur*” OR “self inflicted injur*” OR suicid* OR “para-suicid*” OR parasuicid* OR “suicidal behav*” OR “self?harm*” OR “self?injur*” OR “self?poison*” OR “self?inflict*”)

#2

TS= (“pathological Internet use” OR “Problematic Internet use” OR “Internet addiction” OR “Internet Addiction Disorder” OR “internet use” OR “gaming addiction” OR “internet gaming disorder” OR “Mobile phone addiction” OR “Smartphone addiction”)

#3

TS=(”cell phone” OR “cell phones” OR “cellular phone” OR “cellular phones” OR “cellular telephone” OR “cellular telephones” OR “mobile devices” OR “mobile phone” OR “smart phone” OR “smartphone”)

#4

TS=(addiction OR dependence OR dependency OR abuse OR “addicted to” OR overuse OR “problem use” OR “compensatory use”)

#5

#1 AND (#2 OR (#3 AND #4)) AND LANGUAGE: (English)

N=529

***EMBASE***

#1

'deliberate self harm'/exp OR 'self injurious behavior'/exp OR 'self-injury'/exp

#2

'self injurious behavior':ab,ti OR 'self-injury':ab,ti OR 'self-harm':ab,ti OR 'self harm*':ab,ti OR 'self-injur*':ab,ti OR 'self injur*':ab,ti OR 'self-cut*':ab,ti OR 'self-destruct':ab,ti OR 'nonsuicidal self-injur*':ab,ti OR 'non-suicidal self injur*':ab,ti OR 'deliberate self harm':ab,ti OR 'self-mutil*':ab,ti OR overdos*:ab,ti OR 'self-inflicted injur*':ab,ti OR 'self inflicted injur*':ab,ti OR suicid*:ab,ti OR 'para-suicid*':ab,ti OR parasuicid*:ab,ti OR 'suicidal behav*':ab,ti OR 'self?harm*':ab,ti OR 'self?injur*':ab,ti OR 'self?poison*':ab,ti OR 'self?inflict*':ab,ti

#3

'internet addiction disorder'/exp

#4

'pathological internet use':ab,ti OR 'problematic internet use':ab,ti OR 'internet addiction':ab,ti OR 'internet addiction disorder':ab,ti OR 'internet use':ab,ti OR 'gaming addiction':ab,ti OR 'internet gaming disorder':ab,ti OR 'mobile phone addiction':ab,ti OR 'smartphone addiction':ab,ti

#5

'cell phone':ab,ti OR 'cell phones':ab,ti OR 'cellular phone':ab,ti OR 'cellular phones':ab,ti OR 'cellular telephone':ab,ti OR 'cellular telephones':ab,ti OR 'mobile devices':ab,ti OR 'mobile phone':ab,ti OR 'smart phone':ab,ti OR 'smartphone':ab,ti

#6

addiction:ab,ti OR dependence:ab,ti OR dependency:ab,ti OR abuse:ab,ti OR 'addicted to':ab,ti OR overuse:ab,ti OR 'problem use':ab,ti OR 'compensatory use':ab,ti

#7

(#1 OR #2) AND (#3 OR #4 OR (#5 AND #6)

#8

limit to (human and (article or article in press)

N=466

**Supplementary material 2:** **Methodological quality assessment of the literature**

S2a： Agency for Health Care Research and Quality (AHRQ)

| Study | Year | ① | ② | ③ | ④ | ⑤ | ⑥ | ⑦ | ⑧ | ⑨ | ⑩ | ⑪ |
| --- | --- | --- | --- | --- | --- | --- | --- | --- | --- | --- | --- | --- |
| Lam et al | 2009 | Y | Y | Y | Y | U | N | Y | N | N | Y | N |
| Duan et al | 2013 | Y | Y | Y | Y | U | N | Y | Y | N | Y | N |
| Tang et al | 2016 | Y | Y | Y | Y | U | N | Y | Y | N | N | N |
| Huang et al | 2016 | Y | Y | Y | Y | U | Y | N | N | N | Y | N |
| Liu et al | 2017 | Y | Y | Y | Y | U | N | Y | N | N | Y | N |
| Cao et al | 2019 | Y | Y | Y | Y | U | N | N | N | N | Y | N |
| Li et al | 2019 | Y | Y | Y | Y | U | N | N | N | N | Y | N |
| Pang and Wang | 2020 | Y | Y | Y | Y | U | Y | N | Y | N | N | N |
| Tang et al | 2020 | Y | Y | Y | Y | U | Y | Y | Y | N | Y | N |
| Wang et al | 2022 | Y | Y | Y | Y | U | Y | Y | Y | N | Y | N |
| Qian et al | 2022 | Y | Y | Y | Y | U | Y | N | N | N | Y | N |
| Rong et al | 2023 | Y | Y | Y | Y | U | Y | Y | Y | N | Y | N |

Y: Yes; N:No; U: Uclear; ①Define the source of information (survey, record review); ②List inclusion and exclusion criteria for exposed and unexposed subjects(cases and controls) or refer to previous publications; ③Indicate time period used for identifying patients; ④indicate whether or not subjects were consecutive if not population-based; ⑤indicate if evaluators of subjective components of study were masked to other aspects of the status of the participants; ⑥Describe any assessments undertaken for quality assurance purposes(e.g., test/retest of primary outcome measurements); ⑦Explain any patient exclusions from analysis; ⑧Describe how confounding was assessed and/or controlled; ⑨If applicable, explain how missing data were handled in the analysis; ⑩Summarize patient response rates and completeness of data collection; ⑪Clarify what follow-up, was expected and percentage of patients for which incomplete data or follow-up was obtained.

S2b： **Newcastle-Ottawa Scales of recruited studies** (NOS)

| Study | Year | Selection | Comparability | Outcome | Total |
| --- | --- | --- | --- | --- | --- |
| Hsieh et al | 2018 | 2 | 2 | 2 | 6 |
| Pan and Yeh | 2018 | 3 | 2 | 2 | 7 |
| Ma et al | 2023 | 3 | 2 | 3 | 8 |
